# A redox homeostasis disruptor based on a biodegradable nanoplatform for ultrasound (US) imaging-guided high-performance ferroptosis therapy of tumors

**DOI:** 10.1080/14686996.2024.2351354

**Published:** 2024-05-23

**Authors:** Xia Li, Huijian Lin, Jianbo Hu, Jiajin Fang, Hongsheng Liu, Can Fu, Kewei Zhao

**Affiliations:** a Functional Examination Department, The Third Affiliated Hospital of Guangzhou University of Chinese Medicine, Guangzhou, Guangdong, China; b Medical Imaging Department, The Third Affiliated Hospital of Guangzhou University of Chinese Medicine, Guangzhou, Guangdong, China; c Science Experiment Center, Guangdong Huayan Biomedical Technology Centre, Guangzhou, China; d Laboratory Department, The Third Affiliated Hospital of Guangzhou University of Chinese Medicine, Guangzhou, Guangdong, China

**Keywords:** Hollow mesoporous organosilica nanoparticle, ferroptosis therapy, CO bubble, ultrasound imaging, tumor microenvironment

## Abstract

The synergistic disruption of intracellular redox homeostasis through the combination of ferroptosis/gas therapy shows promise in enhancing the antitumor efficacy. However, the development of an optimal delivery system encounters significant challenges, including effective storage, precise delivery, and controlled release of therapeutic gas. In this study, we propose the utilization of a redox homeostasis disruptor that is selectively activated by the tumor microenvironment (TME), in conjunction with our newly developed nanoplatforms (MC@HMOS@Au@RGD), for highly efficient ferroptosis therapy of tumors. The TME-triggered degradation of HMOS initiates the release of MC and AuNPs from the MC@HMOS@Au@RGD nanoplatform. The released MC subsequently reacts with endogenous hydrogen peroxide (H_2_O_2_) and H^+^ to enable the on-demand release of CO gas, leading to mitochondrial damage. Simultaneously, the released AuNPs exhibit GOx-like activity, catalyzing glucose to generate gluconic acid and H_2_O_2_. This process not only promotes the decomposition of MnCO to enhance CO production but also enhances the Fenton-like reaction between Mn^2+^ and H_2_O_2_, generating ROS through the modulation of the H^+^ and H_2_O_2_-enriched TME. Moreover, the generation of CO bubbles enables the monitoring of the ferroptosis treatment process through ultrasound (US) imaging. The efficacy of our prepared MC@HMOS@Au@RGD disruptors in ferroptosis therapy is validated through both *in vitro* and *in vivo* experiments.

## Impact Statement

A strategy of disrupted redox homeostasis specifically initiated by the tumor microenvironment and our constructed MC@HMOS@Au@RGD nanoplatforms is proposed for ultrasound (US) imaging-guided potent ferroptosis therapy of tumors.

## GRAPHICAL ABSTRACT

**Figure:**
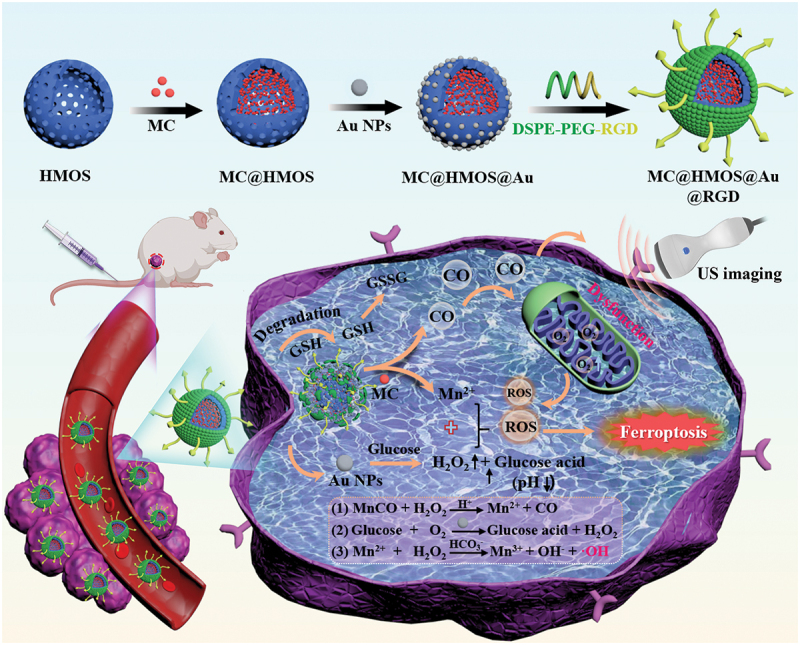

## 1. Introduction

Ferroptosis is a novel form of programmed cell death that relies on iron and is distinct from apoptosis, cellular necrosis, and autophagy [1–3]. In ferroptosis, the oxidation of unsaturated fatty acids, abundant in cell membranes, is catalyzed by divalent iron or ester oxygenase, leading to lipid peroxidation and subsequent cell death [4–6]. This unique mechanism holds great promise in tumor treatment due to its ability to selectively target drug-resistant cells and tumor-specific vulnerabilities [7]. To induce more effective ferroptosis, a range of iron-based nanomaterials, including Fe-DNA/GOx@ZIF-8 [8], FePt@MnO [9], Fe/Fe_3_O_4_ [10], FePt/MoS_2_ [11], PFP@Fe/Cu-SS [12], and FeOOH [13], have been developed. However, the presence of a robust intracellular defense system, characterized by the overproduction of reactive oxygen species (ROS) that can be counteracted by the excessive reductive glutathione (GSH) in tumor cells, hinders the efficiency of ferroptosis therapy [14]. Consequently, overcoming the redox homeostasis of tumor cells represents a critical challenge in achieving effective tumor treatment through ferroptosis and holds significant research implications.

To address the aforementioned limitations in tumor ferroptosis therapy, numerous designs of catalytic nanomedicines have been developed to enhance the ferroptosis of tumor cells through efficient H_2_O_2_ self-supply or GSH scavenging [15–17]. The enhancement of ferroptosis therapy efficacy through these approaches is noteworthy; however, it remains constrained by the comparatively sluggish pace of the Fenton reaction. Notably, relying solely on ferroptosis as a single modality poses challenges in achieving effective cancer treatment. This is primarily attributed to the inadequate generation of toxic ROS by low-dose Fenton reagents for tumor cell eradication, while high-dose Fenton reagents lead to undesirable side effects on normal cells and tissues [18,19]. As a result, there is a pressing and imperative need to explore alternative, yet efficient and safe, cooperative strategies for cancer therapy.

As an environmentally friendly treatment approach, carbon monoxide (CO) gas therapy has demonstrated significant potential in inducing mitochondrial dysfunction by elevating ROS levels in cancer cells [20,21]. thereby directly suppressing tumor growth. Consequently, CO-based gas therapy has emerged as a promising therapeutic modality in recent years. However, there are two primary challenges that impede the application of CO in cancer therapy. Firstly, the poor tumor-targeting ability of CO, coupled with its strong affinity for hemoglobin, increases its toxicity to the human body [22]. Secondly, the development of an ideal delivery system for efficient storage, targeted delivery, and controlled release of CO remains a critical concern [23].

Among the various emerging nanocarriers, hollow mesoporous organosilica nanoparticles (HMONs) have garnered significant attention as drug delivery vehicles [24]. This is primarily due to their minimal systemic toxicity, unique biodegradability, tunable mesopore size, and large storage capacity for diverse therapeutic agents [25]. However, the unexpected premature release of drugs from the pore channels of HMONs during blood circulation poses uncontrolled biosafety risks [26]. To address this issue, anchor gatekeeper strategies can be employed to impede drug leakage and enhance loading capacity [27]. For example, Huang et al. employed core/shell Fe_3_O_4_/Gd_2_O_3_ hybrid nanoparticles with high contrast MRI functionality as gatekeepers to seal HMONs pores, enhancing drug delivery efficiency and enabling tumor-specific drug release [28]. Guo et al. developed a novel approach by incorporating co-loaded platinum nanoparticles (Pt-NPs) and IR820 (a photosensitizer) within HMONs to enhance the efficacy of immunotherapy in gastric cancer [29]. The gatekeepers attached to HMONs bring along the combined therapeutic and/or imaging functions, along with a substantial enhancement in preventing early drug release [30–32], which highlighting the promising potential for merging tumor diagnosis and treatment. Nonetheless, a significant challenge lies in identifying suitable gatekeepers that effectively match the mesopores and possess collaborative theranostic functions for on-demand drug release in tumors. Finding gatekeepers that fulfill these requirements remains a complex task.

**Scheme 1.**
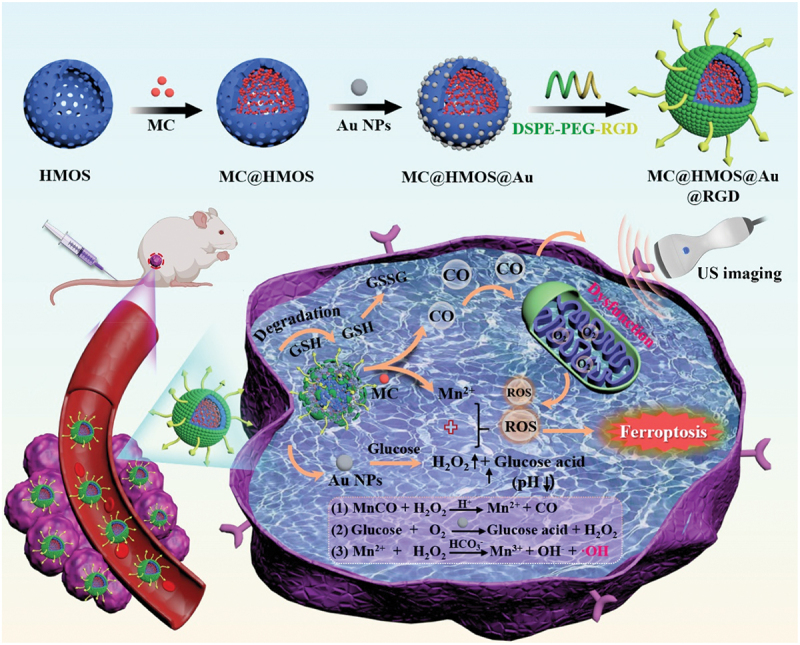
(a) Schematic illustration for the construction of MC@HMOS@Au@RGD nanoplatform. (b). Schematic illustration of the MC@HMOS@Au@RGD with US self-navigation and destruction of redox homeostasis for high-performance ferroptosis therapy.

In this study, a non-iron nanoplatform was developed as a disruptor of redox homeostasis for high-performance ultrasound (US) imaging-guided ferroptosis therapy. The nanoplatform consisted of a carbon monoxide (CO) prodrug, MnCO (MC), loaded into sulfhydryl-modified hollow mesoporous organosilica nanoparticles (HMOS) with blocked pores using ultra-small gold nanoparticles (AuNPs). The resulting MC@HMOS@Au nanoplatform was further coated with a peptide called Arg-Gly-Asp (RGD) that was decorated with distearoyl-sn-glycerol-3-phosphoethanolamine-N-[methoxy (polyethylene glycol)-2000] (DSPE-PEG-RGD), generating the MC@HMOS@Au@RGD nanoplatform (Scheme 1(a)). As illustrated in Scheme 1(b), the MC@HMOS@Au@RGD nanoplatform could accumulate in tumor tissues through the enhanced permeability and retention (EPR) effect and internalize into tumor cells *via* integrin α_v_β_3_-mediated endocytosis. Once inside the cells, the reductive GSH present in the tumor microenvironment (TME) would initiate the degradation of HMOS, leading to the specific release of MC and AuNPs from the nanoplatform. The released MC would then react with endogenous H_2_O_2_ and H^+^ to trigger the on-demand release of CO gas, resulting in mitochondrial damage. Simultaneously, the released AuNPs, which exhibited glucose oxidase-like activity, would catalyze glucose to produce gluconic acid and H_2_O_2_. This process not only enhanced the decomposition of MnCO to produce more CO but also intensified the Fenton-like reaction between Mn^2+^ and H_2_O_2_, generating ROS in the TME, which was enriched with H^+^ and H_2_O_2_.

Furthermore, the generation of CO bubbles allowed the monitoring of the ferroptosis treatment process using ultrasound (US) imaging. The effectiveness of this approach was evaluated both *in vitro* and *in vivo*. The strategy developed in this study integrates ferroptosis/gas therapy to disrupt intracellular redox homeostasis, thereby amplifying the effectiveness of antitumor treatments. Conquering hurdles in optimal delivery systems, including storage, precise delivery, and controlled gas release, is imperative for success. This innovative approach signifies a promising pathway for advancing targeted and efficient tumor therapy through precise redox modulation and gas-mediated interventions.

## 2. Results and discussion

### 2.1. Synthesis and characterization of nanoplatform

**Figure 1.**
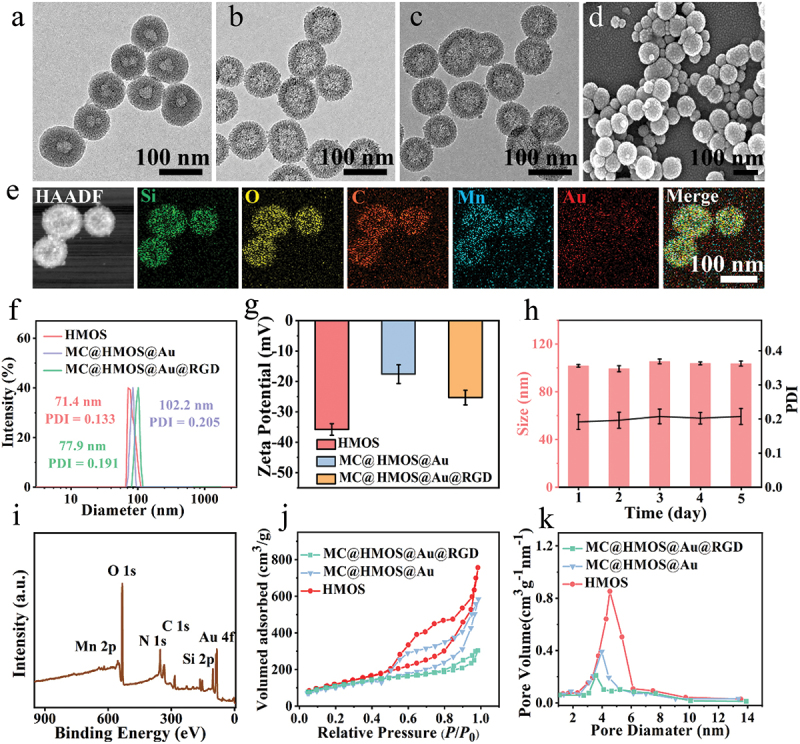
(a-c) TEM images of HMOS (a), HMOS@Au (b), and MC@HMOS@Au@RGD (c). (d) SEM image of obtained MC@HMOS@Au@RGD. (e) HAADF images of MC@HMOS@Au@RGD and corresponding element mapping images of Si, O, C, Mn, and Au. (f, g) Size distributions (f) or zeta potentials (g) measured by DLS. (h) Hydrodynamic particle size and polydispersity index (PDI) of MC@HMOS@Au@RGD in PBS (pH 7.4) buffer within 5.0 days. (i) Full range XPS spectra of MC@HMOS@Au@RGD. (j, h) Pore size distributions (j), or N_2_ adsorption-desorption isotherms (k) of various nanoparticles.

Figure 1(a) demonstrates the fabrication of HMOS with a uniform spherical morphology and hollow structure. This was achieved by modifying HMONs with 3-mercaptopropyltrimethoxysilane (MPTMS). To prevent premature drug leakage and enhance drug loading capacity, ultrasmall AuNPs with glucose oxidase (GOx)-mimic activity were used as anchor gatekeepers on the pore channels of HMOS. Figure 1(b) illustrates that the ultrasmall AuNPs (~3.0 nm) were well dispersed and immobilized within the mesopore channels of HMOS, forming HMON@Au nanoplatforms through Au-S bonds. This anchoring of AuNPs within the mesopores of HMOS effectively prevented drug leakage. The MC@HMOS@Au@RGD nanoplatform was obtained through a series of functionalization steps, including the encapsulation of the CO prodrug (MC) into the cavities of HMOS, anchoring of AuNPs within the mesopores, and coating of DSPE-PEG-RGD onto the surface of HMOS. The resulting nanoplatform (*i.e*., MC@HMOS@Au@RGD) exhibits excellent MC loading content, reaching 15.6%. TEM images of MC@HMOS@Au@RGD (Figure 1(c)) show uniform spherical morphologies and respectable monodispersity, indicating that the aforementioned functionalization steps did not significantly affect the morphology and dispersion of the nanoplatforms. This observation was consistent with the scanning electron microscopy (SEM) image shown in Figure 1(d).

The chemical compositions of MC@HMOS@Au@RGD were analyzed using scanning transmission electron microscopy (STEM) mapping, as shown in Figure 1(e). The mapping displays homogeneous distributions of signals corresponding to Si, O, C, Mn, and Au within the HMOS skeleton. This confirms the successful construction of MC@HMOS@Au@RGD, with all the desired elements present in the nanoplatform. Dynamic light scattering (DLS) analysis was performed to determine the mean hydrodynamic diameters of HMOS, MC@HMOS@Au, and MC@HMOS@Au@RGD, as shown in Figure 1(f). The results reveal that the mean hydrodynamic diameters were approximately 71.4 nm for HMOS, 77.9 nm for MC@HMOS@Au, and 102.2 nm for MC@HMOS@Au@RGD. These measurements indicate an increase in size during the functionalization steps. The corresponding zeta potentials of the nanoplatforms were also measured and are presented in Figure 1(g). The zeta potentials are −35.8 mV for HMOS, −17.6 mV for MC@HMOS@Au, and −25.3 mV for MC@HMOS@Au@RGD. The variations in hydrodynamic size and zeta potentials, as determined by DLS, further confirm the successful preparation of MC@HMOS@Au@RGD. Importantly, due to the high negative charges on the surface of MC@HMOS@Au@RGD, the nanoplatform dispersed in phosphate-buffered saline (PBS) buffer maintained prominent dispersion stability for up to 5.0 days (Figure 1(h)). This stability is crucial for the effective delivery and performance of the nanoplatform in biological systems.

X-ray photoelectron spectroscopy (XPS) analysis of MC@HMOS@Au@RGD (Figure 1(i)) reveals representative peaks corresponding to the Mn 2p, O 1s, N 1s, C 1s, Au 4f, and Si 2p signals. This indicates the presence of Mn from the MC and Au from the AuNPs within the nanoplatform. The presence of these elements confirms the successful incorporation of MC and AuNPs into the HMOS framework. The Brunauer-Emmett-Teller (BET) evaluations were conducted to determine the specific surface area and pore size of the nanoplatforms. HMOS exhibited a large specific surface area of 456.3 m^2^ g^−1^ (Figure 1(j)) and a pore size of approximately 4.5 nm (Figure 1(k)). However, after the functionalization steps involving the anchoring of AuNPs and the coating of DSPE-PEG-RGD, the specific surface area and pore size of T MC@HMOS@Au or MC@HMOS@Au@RGD decrease to 371.2 or 174.3 m^2^ g^−1^, and approximately 3.9 or 3.4 nm, respectively. These results indicate the successful anchoring of AuNPs as gatekeepers and the coating of DSPE-PEG-RGD onto the nanoplatform, which led to a reduction in the specific surface area and pore size.

### 2.2. In vitro verification of the mechanism for redox homeostasis destruction

**Figure 2.**
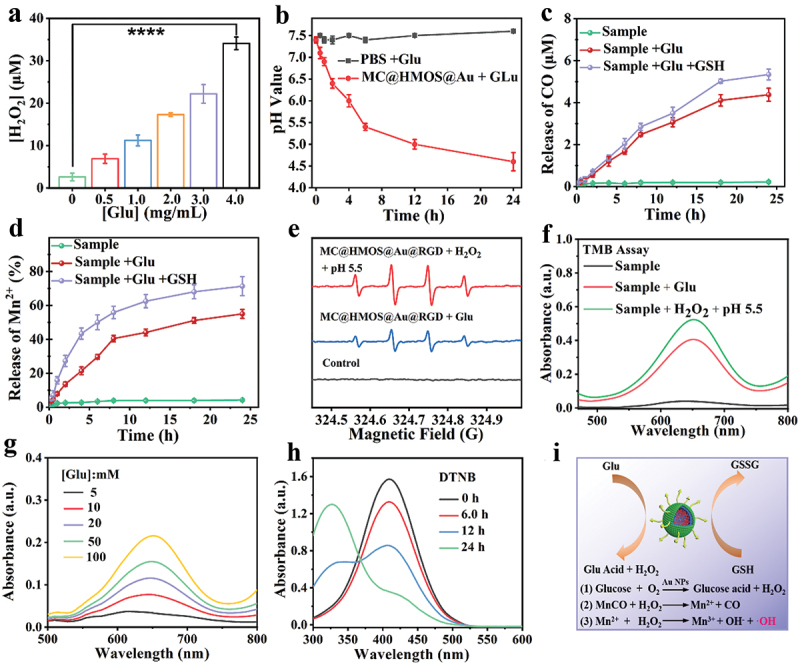
(a) Evaluation of the ability of MC@HMOS@Au@RGD to catalyze the production of H_2_O_2_ by glu. (b) The pH values of the MC@HMOS@Au@RGD reacted with glucose at various intervals. (c, d) Cumulative release behavior of CO or Mn^2+^ in different incubation conditions. (e) ESR spectra of various groups for the detection of •OH radical. Data are presented as means ± SD (*n* = 3). (f) UV-vis absorption spectra of TMB after treatment with MC@HMOS@Au@RGD in different conditions. (g) Glucose dependence of •OH generation based on TMB assays. (h) Time-dependent GSH scavenging performance of MC@HMOS@Au@RGD with DTNB as a probe of -SH. (i) Schematic illustration of •OH scavenging mechanism based on the SOD-like and CAT-like capabilities of the KGN@HMZC@HA nanozyme. Statistical significance was analyzed by a one-way ANOVA. ****p < 0.0001.

The successful fabrication of the nanoplatforms prompted us to investigate their catalytic functions in a systematic manner. It is well-known that AuNPs exhibit GOx-like activity, catalyzing the conversion of glucose into gluconic acid and H_2_O_2_ in the presence of oxygen [33]. Figure 2(a) demonstrates the glucose dependence of H_2_O_2_ generation performance based on the GOx-like activity of AuNPs. Compared to the control group, the evident increase of H_2_O_2_ concentration is observed in Glu group with 4.0 mg/mL (****p < 0.0001). In line with this, the acidity of the MC@HMOS@Au@RGD solution incubated with glucose increases over time, as shown in Figure 2(b). This time-dependent increase in acidity is attributed to the catalysis of glucose by the anchored AuNPs within the HMOS mesoporous structure, which simultaneously produces H^+^ and H_2_O_2_.

When the MC@HMOS@Au@RGD group is exposed to glucose and 5.0 mM of GSH within 24 h, a further elevated release of CO or Mn^2+^ is observed (Figure 2(c,d). This can be attributed to the degradation of HMOS in response to GSH, which enhances the GOx-like activity of AuNPs, leading to a more efficient release of CO and Mn^2+^. Overall, these findings demonstrate the catalytic capabilities of the MC@HMOS@Au@RGD nanoplatform, particularly in the presence of glucose and GSH, resulting in the generation of CO and Mn^2+^ and highlighting its potential for therapeutic applications.

The electron spin resonance (ESR) spectra provide further evidence of the catalytic activity of MC@HMOS@Au@RGD in generating hydroxyl radicals (•OH). When MC@HMOS@Au@RGD is mixed with glucose or H_2_O_2_ under acidic conditions, significant amounts of •OH are rapidly generated, as indicated by the characteristic 1:2:2:1 signal intensities in Figure 2(e). Similar results are observed in the 3,3’,5,5’-tetramethylbenzidine (TMB) assay. Figure 2(f) shows that MC@HMOS@Au@RGD at neutral pH (pH 7.4) exhibits negligible absorption at 652 nm. However, in the presence of glucose or H_2_O_2_ under acidic conditions (pH 5.5), the distinctive absorption of TMB gradually increases for the sample + glucose or sample + H_2_O_2_ group. This indicates the generation of reactive species capable of oxidizing TMB. Furthermore, the glucose dependence of •OH productivity is demonstrated in Figure 2(g). The results indicate that the presence of glucose enhances the production of •OH by MC@HMOS@Au@RGD. This observation further supports the GOx-like capability of the nanoplatform, which facilitates a favorable Fenton-like catalytic activity between Mn^2+^ and H_2_O_2_.

The presence of disulfide (S-S) bonds in MC@HMOS@Au@RGD suggests that the nanoplatform has the potential to consume GSH. To investigate this, the 5,5’-dithio-bis-2-(nitrobenzoic acid) (DTNB) probe was used to measure the GSH consumption capability of MC@HMOS@Au@RGD through UV-vis spectra. Figure 2(h) demonstrates that the absorption peak of DTNB at around 412 nm decreases significantly with increasing incubation time after incubation with GSH and MC@HMOS@Au@RGD. This observation indicates a remarkable time dependence of the GSH depletion abilities of the MC@HMOS@Au@RGD nanoplatforms.

Consequently, the simultaneous generation of •OH and consumption of GSH by MC@HMOS@Au@RGD play a significant role in the accumulation of ROS for cancer treatment through a cascade catalysis procedure, as depicted in Figure 2(i). The concurrent generation of •OH and GSH consumption by MC@HMOS@Au@RGD is highly conducive to the accumulation of ROS, which can be utilized for cancer treatment through a cascade catalysis procedure.

### 2.3. Cellular uptake and lysosome escape of the nanoplatforms

**Figure 3.**
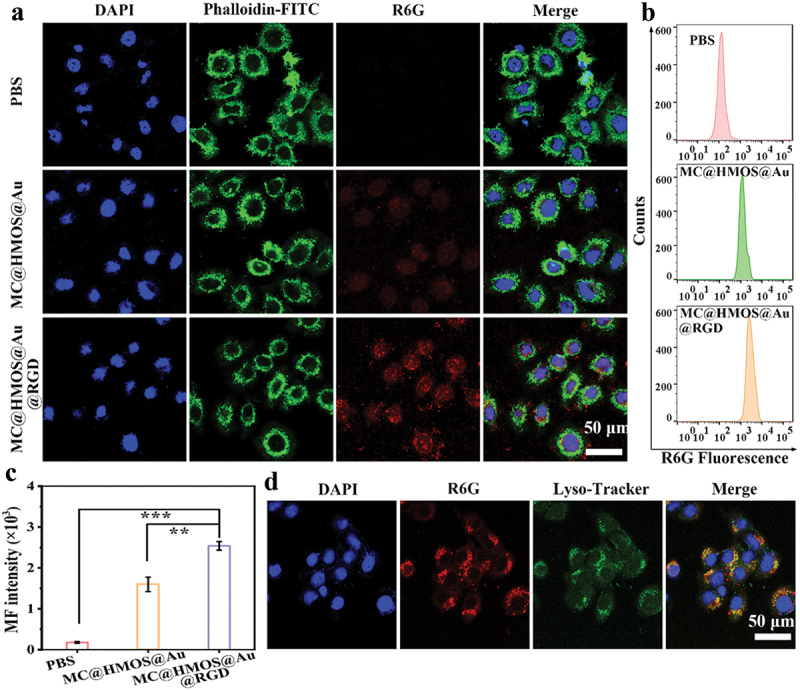
(a) CLSM detection of the cell uptake behavior of R6G-labelled MC@HMOS@Au or MC@HMOS@Au@RGD. (b) Fluorescence distributions and (c) corresponding quantitative analysis of 4T1 cells were measured by flow cytometry after different treatments. (d) CLSM images of lysosome-stained 4T1 cells exposed to R6G-labelled MC@HMOS@Au@RGD for 6.0 h. Statistical significance was analyzed by a one-way ANOVA. **p < 0.01 and ***p < 0.001.

The intracellular endocytosis performance of MC@HMOS@Au or MC@HMOS@Au@RGD was assessed in α_v_β_3_-positive 4T1 cells using confocal laser scanning microscopy (CLSM) and flow cytometry. CLSM observations revealed that 4T1 cells treated with nanoplatforms without RGD decoration exhibited a very low red fluorescence signal of R6G. In contrast, a significantly increased cellular uptake of the RGD-conjugated nanoplatforms was observed in the integrin α_v_β_3_-positive 4T1 cells (Figure 3(a)). Flow cytometry results (Figure 3(b,c)) further supported these findings, showing that the fluorescence intensities of the R6G-labeled MC@HMOS@Au@RGD group were much stronger compared to the group treated with R6G-labeled MC@HMOS@Au (**p < 0.01). Obviously, the incorporation of RGD decoration on the nanoplatforms led to increased cellular uptake in α_v_β_3_-positive 4T1 cells. The specific recognition between the RGD ligand and integrin α_v_β_3_ facilitated the enhanced intracellular endocytosis of the R6G-labeled MC@HMOS@Au@RGD nanoplatforms [34].

In Figure 3(d), CLSM images of lysosome-stained 4T1 cells with Lyso-TrackerTM (green) are shown after incubation with R6G-labeled MC@HMOS@Au@RGD for 6.0 h. The images reveal the presence of distinct separation of red dots from the green fluorescence domain in the nanoplatform groups. This observation indicates the positive lysosomal escape of MC@HMOS@Au@RGD, which is a crucial prerequisite for subsequent ferroptosis therapy of cancer.

### 2.4. Mechanism and efficiency of in vitro anti-tumor therapy

**Figure 4.**
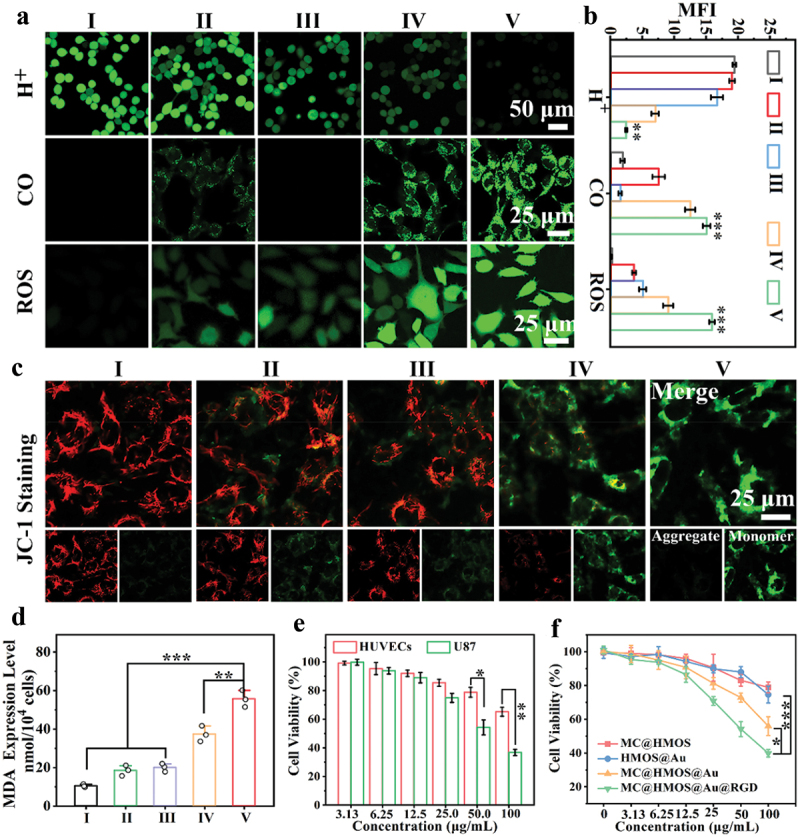
(a) CLSM images indicating intracellular H^+^, CO, or ROS level in 4T1 cells treated with (I) PBS, (II) MC@HMOS, (III) HMOS@Au, (IV) MC@HMOS@Au or (V) MC@HMOS@Au@RGD. (b) Fluorescence quantitative statistics corresponding to H^+^, CO, or ROS. (c) JC-1 staining of 4T1 cells with the formulations of I-V. (d) Quantitative analysis of intracellular MDA level after treatment with the formulations of I-V. (e) Cytotoxicity evaluation of MC@HMOS@Au@RGD, HUVECs (α_v_β_3_-negative expression), or U87 (α_v_β_3_-positive expression) cells. (f) Cell viabilities of 4T1 cells subjected to the MC@HMOS, HMOS@Au, MC@HMOS@Au or MC@HMOS@Au@RGD treatment, respectively. Statistical significance was analyzed by a one-way ANOVA. *p < 0.05, **p < 0.01 and ***p < 0.001.

To evaluate the ability of different formulations to enhance the acidic TME, the variations in tumor intracellular pH were measured using a pH-sensitive probe called 2’,7’-bis-(2-carboxyethyl)-5-(and-6)-carboxyfluorescein, acetoxymethyl ester (BCECF-AM) [35]. The fluorescence of BCECF-AM decreases with an increase in acidity. CLSM observations in Figure 4(a) show the intracellular pH variations in 4T1 cells treated with different formulations. The first row of images, comparing cells treated with PBS or MC@HMOS, reveals no significant difference in intracellular pH. However, a slight decrease in green fluorescence is observed in cells treated with HMOS@Au or MC@HMOS@Au, and a noticeable decrease in green fluorescence is observed in cells treated with MC@HMOS@Au@RGD.The decrease in green fluorescence indicates the acidification of the intracellular environment, which is caused by the catalytic activity of GOx-like ultrasmall AuNPs present in the formulations. The second row of images in Figure 4(a) demonstrates that cells treated with MC@HMOS@Au@RGD exhibit a higher fluorescence intensity compared to cells treated with other formulations. This suggests that MC@HMOS@Au@RGD has a prominent capability to generate CO due to the H_2_O_2_-mediated activation of MC. These results indicate that MC@HMOS@Au@RGD treatment leads to a more pronounced acidification of the intracellular environment and a higher generation of CO compared to other formulations. This validates the capability of MC@HMOS@Au@RGD to potentiate the acidic TME, which is crucial for its subsequent therapeutic effects, such as ferroptosis induction.

To investigate the intracellular therapeutic mechanism of MC@HMOS@Au@RGD, the generation of ROS in tumor cells was measured using 2’,7’-dichlorofluorescein diacetate (DCFH-DA). The third row of images in Figure 4(a) and (b) show the intracellular DCF fluorescence intensity in different treatment groups. The results demonstrate that the RGD-decorated nanomedicine group (MC@HMOS@Au@RGD) exhibits significantly higher intracellular DCF fluorescence intensity compared to other groups. Specifically, the DCF fluorescence intensity in the MC@HMOS@Au@RGD group is 4.3-fold higher than that in the MC@HMOS group, 3.2-fold higher than that in the HMOS@Au group, and 1.8-fold higher than that in the MC@HMOS@Au group. The enhanced ROS generation in the MC@HMOS@Au@RGD group can be attributed to two factors. Firstly, the nanoplatform MC@HMOS@Au@RGD possesses a GOx-like capability, which enables favorable Fenton-like catalytic activity between Mn^2+^ and H_2_O_2_, leading to ROS production. Additionally, previous studies have shown that CO-mediated gas therapy can activate the mitochondrial ROS signaling pathway, further elevating the intracellular ROS levels [36]. Therefore, the activation of the mitochondrial ROS signaling pathway by CO gas therapy, in combination with the Fenton-like catalytic activity, contributes to the amplified ROS cascade observed in tumor cells treated with MC@HMOS@Au@RGD. Taken together, these findings indicate that MC@HMOS@Au@RGD possesses the capability to enhance and amplify the ROS cascade within tumor cells, which is a crucial aspect of its intracellular therapeutic mechanism.

To assess the mitochondrial status of the nanoplatform, the mitochondrial membrane potential (MMP) was evaluated using a JC-1 probe. JC-1 aggregates emit red fluorescence in healthy mitochondria, while JC-1 monomers exhibit green fluorescence in mitochondria with depolarized MMP, indicating mitochondrial dysfunction [37]. Figure 4(c) shows that the highest intensity of green fluorescence is observed in cells treated with MC@HMOS@Au@RGD, indicating the most significant mitochondrial dysfunction among the different treatment groups. This suggests that MC@HMOS@Au@RGD contributes to mitochondrial dysfunction by releasing CO and amplifying ROS levels. To further validate these findings, the malondialdehyde (MDA) assay was performed on 4T1 cancer cells after different treatments (Figure 4(d)). MDA is a marker of lipid peroxidation, and its level can indicate the extent of oxidative damage [38]. The results demonstrate that the MDA level is remarkably elevated after treatment with MC@HMOS@Au@RGD, indicating increased lipid peroxidation. The highest level of lipid peroxidation corresponds to the most severe ferroptosis status observed in the same group. Collectively, the evaluation of mitochondrial membrane potential using the JC-1 probe and the MDA assay both support the conclusion that MC@HMOS@Au@RGD induces mitochondrial dysfunction, amplifies ROS levels and leads to elevated lipid peroxidation.

In Figure 4(e), it is shown that MC@HMOS@Au@RGD exhibits higher toxicity on α_v_β_3_-positive U87 cells compared to αα_v_β_3_-negative HUVECs cells. This is attributed to the difficulty in achieving effective endocytosis of nanoplatforms in cells with low expression of integrin α_v_β_3_. Therefore, the high-performance ferroptosis therapy is specifically applicable to α_v_β_3_-overexpressing cancer cells.

Furthermore, the concentration-dependent cytotoxicity of various nanomedicines against 4T1 cancer cells was observed in Figure 4(f). As expected, the MC@HMOS@Au@RGD-treated group shows the lowest cell viability compared to all control groups, indicated by the asterisks (*p < 0.05 or ***p < 0.001). These results demonstrate that the higher cytotoxicity of MC@HMOS@Au@RGD can be attributed to the augmented ferroptosis, which is facilitated by the α_v_β_3_-mediated active targeting and disruption of intracellular redox balance.

### 2.5. Enhanced US imaging and in vivo biodistribution

**Figure 5.**
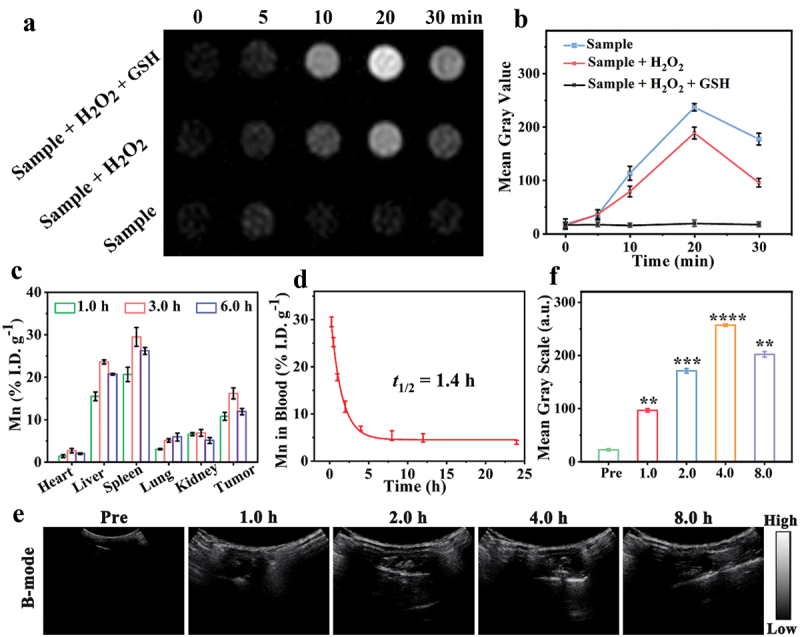
(a) US images of MC@HMOS@Au@RGD incubated with different mediums at different time intervals. (b) Quantitative contrast means the power of corresponding ultrasound images. (c) The pharmacokinetics of MC@HMOS@Au@RGD in mice are expressed as Gd percentage of injected dose per gram of blood (% I.D. g^−1^). (d) Biodistribution of Mn in prime organs and tumors of mice intravenously injected with MC@HMOS@Au@RGD at 1.0, 3.0, or 6.0 h. (e) *In vivo* US images and (f) the corresponding gray values of the mice obtained 8 h after intravenous injection of MC@HMOS@Au@RGD. Statistical significance was analyzed by a one-way ANOVA. *p < 0.05, **p < 0.01, ***p < 0.001 and ****p < 0.0001.

Figure 5a presents ultrasound images of MC@HMOS@Au@RGD under different incubation conditions. In comparison to the pure sample group, the MC@HMOS@Au@RGD with H_2_O_2_ group shows an enhancement of the US signal at various time intervals due to the generation of CO bubbles. Notably, the further degradation of the HMOS framework by GSH leads to a faster and increased generation of CO, resulting in the highest US signal intensities observed in the MC@HMOS@Au@RGD + H_2_O_2_ + GSH group. Figure 5(b) demonstrates the quantitative analysis of grayscale values obtained from the ultrasound images, which also confirms similar outcomes. These results indicate that the addition of H_2_O_2_ and GSH enhances the US signal intensity, reflecting the generation of CO bubbles and the degradation of the HMOS framework.

In Figure 5(c), the blood circulation half-life of MC@HMOS@Au@RGD is determined to be 1.4 hours after intravenous (*i.v*.) injection. This indicates that the nanoplatforms remain in the bloodstream for a relatively extended period before being cleared. To further confirm the tumor enrichment of MC@HMOS@Au@RGD nanoplatforms, the *in vivo* distribution of Mn was studied by analyzing the Mn levels in primary organs or tumors using inductively coupled plasma optical emission spectrometry (ICP-OES). The results show that the highest accumulation of Mn in tumor tissues (16.2 ± 1.3% I.D. g^−1^) occurs at 3.0 h post-injection of MC@HMOS@Au@RGD. Subsequently, the Mn content gradually diminishes to 11.9 ± 0.8% I.D. g^−1^ at 6.0 h (Figure 5(d)). Additionally, the nanoplatforms exhibit notable accumulation of Mn in the liver and spleen, but limited presence in the heart and kidneys. This distribution pattern is attributed to the reticuloendothelial system (RES) mediation, a typical occurrence in nanoparticle delivery [39]. These findings provide evidence for the tumor-targeting ability of MC@HMOS@Au@RGD nanoplatforms, as indicated by the preferential accumulation of Mn in tumor tissues. The gradual decrease in Mn content over time suggests the potential clearance of the nanoplatforms from the tumor site.

Figure 5(e)and F depict US images of 4T1 tumor-bearing mice before and after injection of MC@HMOS@Au@RGD (at a dosage of 5.0 mg/kg) at different time intervals, along with the corresponding quantitative analysis of the tumor US images post-injection of the nanoplatforms.

Following the administration of MC@HMOS@Au@RGD, the US signal strength noticeably increases as the injection duration is prolonged. The signal intensity reaches its peak at 4.0 h and gradually subsides by 8 h, indicating the effective enrichment and metabolism of MC@HMOS@Au@RGD at the tumor site. The corresponding quantitative analysis results exhibit a similar trend. The enhanced signal intensity observed in the US images is primarily attributed to the presence of anchored AuNPs in MC@HMOS@Au@RGD, which possess GOx-like activity for H_2_O_2_-mediated activation of MC in the tumor region. This activation leads to the continuous generation of CO bubbles and the sufficient production of echogenic reflectivity, thereby reinforcing the US signal.

### 2.6. In vivo assessments on therapeutic efficacy and biosafety

**Figure 6.**
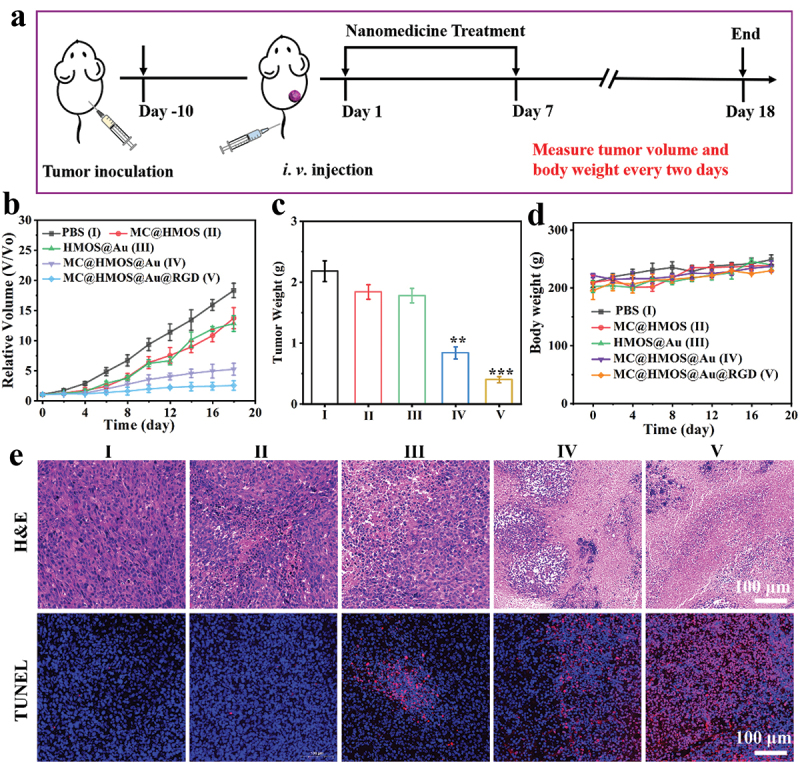
(a) Schematics of intervention in a mouse subcutaneous tumor model. (b-d) Relative tumor volumes (b), tumor weight (c), or body weight records (d) of tumor-bearing mice after treatment with (I) PBS, (II) MC@HMOS, (III) HMOS@Au, (IV) MC@HMOS@Au or (V) MC@HMOS@Au@RGD for 18 days. (e) H&E and TUNEL staining images of tumor tissues after various treatments. Statistical significance was analyzed by a one-way ANOVA. **P < 0.01 and ***P < 0.001.

*In vivo* ferroptosis therapy efficacy was evaluated by intravenous injection of different formulations: (I) PBS, (II) MC@HMOS, (III) HMOS@Au, (IV) MC@HMOS@Au, or (V) MC@HMOS@Au@RGD in 4T1 tumor-bearing mice (Figure 6(a)). The tumor growth curves (Figure 6(b)) and tumor weight on the 18th day (Figure 6(c)) were analyzed to assess the therapeutic effects. The results show that the PBS-treated group exhibited rapid tumor growth. The MC@HMOS and HMOS@Au groups demonstrated partial suppression of tumor growth, indicating some antitumor effect. Notably, the MC@HMOS@Au and MC@HMOS@Au@RGD groups exhibited a significant antitumor effect. In particular, the MC@HMOS@Au@RGD group achieved a tumor inhibition rate of over 90%, indicating the high-performance tumor ferroptosis therapy of MC@HMOS@Au@RGD.

The tumor tissues collected from the sacrificed mice after various treatments were subjected to hematoxylin and eosin (H&E) staining and terminal deoxynucleotidyl transferase dUTP nick end labeling (TUNEL) staining to further investigate the mechanism of antitumor therapy. The staining results (Figure 6(e)) reveal that the MC@HMOS@Au@RGD group exhibits a significant presence of severely necrotic or apoptotic tumor cells. In contrast, the other treatment groups showed relatively low or few numbers of such cells. These results provide further evidence of the high-performance ferroptosis therapy achieved by MC@HMOS@Au@RGD. The active targeting mediated by α_v_β_3_ and the disruption of intracellular redox balance contributes to the observed effects.

Figure S1 demonstrates the low hemolysis percentages (less than 2.0%) of MC@HMOS@Au@RGD at various concentrations. In Figure 6(d), slight weight changes were observed in 4T1 tumor-bearing mice after different treatments over the course of 18 days. Furthermore, the blood routine analysis (Figure S2) and blood biochemical analysis (Figure S3) of healthy mice after intravenous injection of MC@HMOS@Au@RGD showed no noticeable differences in the tested parameters compared to the control group (saline). Additionally, Figure S4 demonstrates that there were no apparent histopathological abnormalities observed in major organs such as the heart, liver, spleen, lung, and kidney in the different treatment groups. Taken together, these results indicate the favorable biosafety of the MC@HMOS@Au@RGD nanoplatform for US-guided high-performance ferroptosis therapy.

## 3. Conclusions

In summary, the developed MC@HMOS@Au@RGD nanoplatforms serve as a disruptor of redox homeostasis, specifically initiated by the TME. The nanoplatforms consist of a CO prodrug, MC, integrated into biodegradable HMOS, with ultrasmall AuNPs acting as gatekeepers to optimize the delivery of MC. To achieve active targeting of tumor cells and prolong the blood circulation time in vivo, DSPE-PEG-RGD is conjugated onto the surface of MC@HMOS@Au, resulting in MC@HMOS@Au@RGD. This nanoplatform is capable of responding to the TME and subsequently remodeling it. Various characterization techniques, such as TEM imaging, particle size and zeta potential measurements using DLS, STEM mapping, BET, and XPS, confirm the successful construction of MC@HMOS@Au@RGD nanoplatforms. Upon endocytosis by tumor cells, MC@HMOS@Au@RGD undergoes rapid biodegradation and elimination of the blocking AuNPs gatekeeper. This releases MC, which reacts with endogenous H_2_O_2_ and H^+^ to achieve on-demand release of CO gas, leading to mitochondrial damage and further generation of ROS. Simultaneously, the released AuNPs exhibit GOx-like activity, catalyzing glucose to produce gluconic acid and H_2_O_2_. This process not only accelerates the decomposition of MC for enhanced CO production but also enhances the Fenton-like reaction activity between Mn^2+^ and H_2_O_2_, generating ROS driven by the remodeling of the acidic and H_2_O_2_-enriched TME. Furthermore, the generation of CO bubbles allows the monitoring of the ferroptosis treatment process through US imaging. The increased acidification, self-supplied H_2_O_2_, and elevated consumption of GSH promote the Fenton-like reaction between Mn^2+^ and H_2_O_2_, resulting in a cyclic catalysis effect. Both *in vitro* and *in vivo* results validate the ferroptosis therapy potential of the prepared MC@HMOS@Au@RGD disruptor. The negligible hemolysis percentages (<2.0%) and the histopathological examination (H&E staining) of major organs indicate the reliable biosafety of MC@HMOS@Au@RGD, making it a promising candidate for precise tumor theranostics.

## Supplementary Material

Supplemental Material

## Data Availability

The data that support the findings of this study are available in the supplementary material of this article.
