# Supplementary material for: A redox homeostasis disruptor based on a biodegradable nanoplatform for ultrasound (US) imaging-guided high-performance ferroptosis therapy of tumors
Source: Sci Technol Adv Mater. 2024 May 23;25(1):2351354. doi: 10.1080/14686996.2024.2351354 (PMC11123443; doi:10.1080/14686996.2024.2351354)
Supplement: Supplemental Material [file TSTA_A_2351354_SM6409.docx]

**Supporting Information**

**A** **redox homeostasis disruptor based on a biodegradable nanoplatform for** **ultrasound (US) imaging-guided high-performance ferroptosis therapy of tumors**

*Xia Li ^1 *^, Huijian Lin ^1^, Jianbo Hu ^2^, Jiajing Fang ^1, 4^, Hongsheng Liu^1, 4^, Can Fu ^1^, Kewei Zhao ^3 *^*

^1^ Functional Examination Department, The Third Affiliated Hospital of Guangzhou University of Chinese Medicine, Guangzhou, Guangdong, China.

^2^ Medical Imaging Department, The Third Affiliated Hospital of Guangzhou University of Chinese Medicine, Guangzhou, Guangdong, China.

^3^ Laboratory Department, The Third Affiliated Hospital of Guangzhou University of Chinese Medicine, Guangzhou, Guangdong, China.

^4^ Guangdong Huayan Biomedical Technology Centre, Guangzhou, China.

*^*^* **Corresponding Author:**

Xia Li Lisayulee2023@outlook.com

Kewei Zhao zkw2011@gzucm.edu.cn

Experimental Section

**Materials:** Bovine serum albumin (BSA), HAuCl_4_, Manganese carbonyl (MnCO), and NaBH_4_ were purchased from Sigma-Aldrich (USA). Cetanecyltrimethylammonium chloride (CTAC, 99.9 %) was obtained from Shanghai Yuanye Biological Technology Co., Ltd. Tetraethylorthosilicate (TEOS), ammonia solution (NH_3_·H_2_O, 25-28 %), triethanolamine (TEA), bis[3-(triethoxysilyl)propyl]tetrasulfide (BTES), sodium citrate (3-mercaptopropyl)-trimethoxysilane (MPTES), glutathione (GSH, 98.0 %), 5,5’-dithiobis-2-(nitrobenzoicacid) (DTNB), 3,3’,5,5’-Tetramethylbenzidine (TMB), and rhodamine6G (R6G) were bought from Shanghai Macklin Biochemical Co., Ltd. Arg-Gly-Asp (RGD) peptide decorated Distearoyl-snglycerol-3-phosphoethanolamine-N-[methoxy (polyethylene glycol)-2000] (DSPE-PEGRGD) was bought from Top peptide Biotech (Shanghai, China). 2’,7’-dichlorofluorescein diacetate (DCFH-DA) were provided from Invitrogen Life Technologies (USA). 2,7-bis(2-carboxyethyl)-5,6-carboxyfluorescein acetoxymethyl ester (BCECF-AM), COP-1 CO fluorescence probe, 5,5’,6,6’-tetrachloro-1,1’,3,3’-tetraethylbenzimidazolylcarbocyanine iodide (JC-1) Assay Kit, MDA Assay Kit, Lyso-tracker red probe, MTT Detection Kit were purchased from Beyotime Biotechnology Co., Ltd. (Shanghai, China).

**Synthesis of HMON:** Monodispersed hollow mesoporous organosilica nanoparticles (HMON) were prepared by a typical “hard template” method. Briefly, 2.0 g of CTAC and 0.10 g of TEA were premixed with 20 mL of Milli-Q water. The solution was then stirred at 80 °C for 30 min, followed by an injection of 1.0 mL of TEOS solution. After 1.0 h, a mixture of silica precursors with 0.8 mL of BTES and 0.4 mL of TEOS was dropwise added into the reaction system, which was stirred for another 4.0 h to obtain core/shell structured mesoporous SiO_2_/organosilica nanoparticles (MSN@MON). The resulting mixture was centrifuged (25000 × g, 15 min) and washed several times with Milli-Q water and ethanol. The CTAC template was then extracted with concentrated HCl and ethanol (v/v = 1:10) at 78 °C for 12 h. To etch the MSNs core, the MSN@MON was redispersed in a 100 mL of aqueous solution containing 0.56 % of NH_3_•H_2_O. After 4.0 h of reaction at 95 °C, the final HMON products were collected through centrifugation (25000 × g, 15 min) and washing with Milli-Q water for several times.

**Synthesis of HMOS:** The as-prepared HMON (15 mg) was dispersed in ethanol (40 mL), followed by addition of a mixture of MPTES (0.15 mL) and ammonia (0.2 mL, 25 wt %) aqueous solution. Then the obtained solution was stirred overnight, and the resultant HMON-SH (*i.e.*, HMOS) was collected after centrifugation and washing with ethanol for next use.

**Synthesis of AuNPs:** 50.0 mg/mL BSA and 5 mM HAuCl_4_ stirred violently. After adjusting to pH12 with 1M NaOH, the solution was stirred for 12 h. AuNP was obtained after dialysis with a dialysis membrane (MW: 20 KD) for 24 h.

**Synthesis of MC@HMOS@Au:** First, MnCO was adsorbed into the mesoporous channel and cavity of HMOP via hydrophobic-hydrophobic interaction. 20 mg of HMOP and 150 μL of MnCO stock solution were added to 15 mL of ethanol for 24 h of stirring. Then the MC@HMOS products were collected by centrifugation, washed with ethanol three times, and dispersed in 20 mL AuNPs dispersions with 3.62 mM of *C*_Au_. After 30 s of vortexing, the mixtures were continuously stirred at room temperature for 6. 0 h. The obtained MC@HMOS@Au was then centrifuged (20000 × g, 10 min), washed three times with pure water, and finally dispersed in 5.0 mL of Milli-Q water. The Au or Mn content in the MC@HMOS@Au was measured by inductively coupled plasma optical emission spectrometry (ICP-OES; Thermo Fisher Scientific, iCAP 6300Duo).

**Synthesis of** **MC@HMOS@Au@RGD:** 2.0 ml of the MC@HMOS@Au dispersions obtained above was centrifuged (20000 × g, 15 min), and then re-dispersed in 2.0 mL of DSPE-PEG2000-RGD aqueous solutions (2.0 mg/mL). After 60 s of vortexing, the mixtures were further stirred for 8.0 h at room temperature, followed by centrifugation (20000 × g, 20 min) and washing with Milli-Q water to obtain MC@HMOS@Au@RGD.

**Characterizations:** The nanoparticle morphologies were acquired from a transmission electron microscope (TEM) (JEM-2100F, JEOL). The size and Zeta potential of nanoparticles were measured using an instrument of dynamic light scattering (DLS) (Nano-Brook 90PlusZata, Brookhaven). Nitrogen absorption-desorption isotherm and porosity were measured by a surface area analyzer (QuadraSorb SI 2000-08, Quantachrome Instruments). X-ray photoelectron spectroscopy (XPS) spectra were obtained using a Thermo Scientific ESCALAB 250 XI.

**GOx-like Catalytic Activity:** To assess the GOx-mimic activity, the content of product H_2_O_2_ was first measured through a spectrophotometric method. Briefly, H_2_O_2_ level in 200 μL aqueous mixture containing sample (100 μg/mL) and glucose (0.5, 1, 2, 3, or 4 mg/mL) was monitored for up to 5 h by using a hydrogen peroxide detection kit (S0038, Beyotime Biotechnology, China). Alternatively, the pH value of 200 μL reaction system containing sample (100 μg/mL) and glucose (1 mM) was measured by using a pH meter at specific intervals.

**Study of Stimulus-Triggered Drug Release:** MC@HMOS@Au@RGD (2.0 mL, 1.0 mg/mL) was sealed into a dialysis bag (cut off *M*_W_: 12 kDa), and then placed in 10 mL of PBS without or with of glucose (20 mM) or GSH (5.0 mM). The dialysis was implemented at 37 ^o^C in an incubator with gently shaking at 100 rpm. At predetermined time points, 0.5 mL of the solution was taken out, and fresh PBS with the same condition and volume was supplemented. The released CO in the PBS was measured by UV-vis spectrophotometer, while the released Mn was quantified by ICP-OES measurement.

**Electron Spin Resonance (ESR) Measurements:** To measure •OH generation *via* ESR, 100 μL of MC@HMOS@Au@RGD (5.0 mg/mL) was incubated with 20 mM glucose or treated with 2.0 mL of acetate buffer (pH 5.5) containing H_2_O_2_ for 6.0 h. After that, 100 μL of 5,5-dimethyl-1-pyrroline-N-oxide (DMPO) was added to the above solution, and the ESR signal was recorded by ESR spectrometer.

**Detection of *In Vitro*** •**OH Production:** 1.0 mL of acetate buffer solution (pH 5.5, 0.8 mM H_2_O_2_) was mixed with MC@HMOS@Au@RGD (40 μL, 2.50-12.5 mg/mL) or that pre-incubated with glucose (20 mM) overnight. The mixtures were kept under stirring at room temperature for 6.0 h. The supernatants were collected by centrifugation at 20000 × g for 10 min, followed by the addition of TMB solution (0.10 mL, 2.0 mM). After 30 min of incubation, the solutions were measured by UV-vis spectroscopy at the wavelength of 652 nm to determine the generation of •OH. The concentration dependence of •OH generation capability for glucose was also studied.

**Detection of GSH Consumption:** To evaluate GSH depletion capability, 200 µL of MC@HMOS@Au@RGD (5.0 mg/mL) was dispersed in GSH solution (5.0 mL,1.0 mM). The mixed solutions were maintained at 37 °C under magnetic stirring for 0, 6.0, 12, or 24 h. The above mixtures were then centrifuged at 20000 × g for 10 min. The obtained supernatants were further mixed with DTNB (50 μL, 10 mg/mL), and the mixtures were incubated for 30 min at 37 °C. After that, the solution was measured by UV-vis spectrophotometer at the wavelength of 412 nm to determine the capability of GSH consumption.

**Cell Culture**: Human glioma U87 cells, Human umbilical vein endothelial cells (HUVECs), or Mouse breast carcinoma (4T1) cells were cultured in the DMEM medium supplemented with 10 % of fetal bovine serum (FBS), 100 U/mL of penicillin G sodium, and 100 μg/mL of streptomycin sulfate. All of the cells were incubated at 37 °C in a humidified atmosphere containing 5.0 % of CO_2_.

**Cellular uptake of R6G-labeled nanoplatform:** R6G (100 μL, 15 mg mL^-1^) was added into a dispersion of MC@HMOS@Au or MC@HMOS@Au@RGD (10 mL, 1.0 mg mL^-1^) and the mixtures were stirred for 24 h in the dark. Then the R6G-labeled MC@HMOS@Au or MC@HMOS@Au@RGD was obtained by centrifugation (15000 × g, 10 min) and washing with deionized water for further use. Uptake of the nanoparticles by 4T1 cells was evaluated *via* confocal laser scanning microscope (CLSM) and flow cytometry, respectively.

For CLSM analysis, 4T1 cells (5.0 × 10^4^ cells) were first seeded into the confocal dishes and allowed to adhere overnight at 37 °C in the normal incubator, followed by culturing under normoxia or hypoxia for another 12 h. Next, the growth media were replaced with a fresh one (0.5 mL) containing R6G-labeled MC@HMOS@Au or MC@HMOS@Au@RGD (10.0 μg/mL of nanoparticles ), and the cells were incubated in a normoxic incubator for 4.0 h. The treated cells were washed with PBS, fixed with 4.0 % of paraformaldehyde, and then the cell nuclei were stained with 4’, 6-diamidino-2-phenylindole (DAPI, Beyotime Biotechnology) for 15 min, followed by treatment with Phalloidin-FITC solution containing 0.1 % of Triton X-100 and 1.0 % of BSA for 20 min. Finally, the cells were observed by a CLSM (Nikon A1-DUVB-2).

For flow cytometry analysis, 4T1 cells were first seeded into six-well plates (2.0 × 10^5^ cells/well), and allowed to adhere overnight at 37 °C. The culture medium was then replaced with a fresh one (1.0 mL) containing R6G-labeled MC@HMOS@Au or MC@HMOS@Au@RGD (10.0 μg/mL of nanoparticles). After incubation for further 4.0 h, the cells were washed twice with cold PBS and harvested by trypsinization, followed by centrifugation (2000 × g, 3.0 min). The obtained cells were re-suspended in PBS and analyzed using flow cytometry (Guava easyCyte, USA).

**Intracellular Lysosome Escape Study:** 4T1 cells (5.0 × 10^4^ cells per well) were seeded into the confocal dish and cultured overnight. The culture media was then replaced with fresh one (0.5 mL) containing MC@HMOS@Au@RGD (*C*_R6G_ = 4.0 μg/mL). After treatment for 6.0 h, the cells were fixed, the nuclei were stained using DAPI for 10 min, and the lysosomes were stained with Lyso-tracker red reagents (50 nM) for 30 min. Finally, the cells were observed by CLSM.

**Monitoring the Treatment-Induced Changes of Intracellular pH Values**: 4T1 cells were seeded into confocal dishes (5.0 × 10^4^ cells per dish) and incubated overnight. Subsequently, the cells were treated with the cells were treated with (I) PBS, (II) MC@HMOS, (III) HMOS@Au, (IV) MC@HMOS@Au, or (V) MC@HMOS@Au@RGD (*C*_nanoparticles_ = 50 μg/mL) for 12 h. The treated cells were then stained with BCECF-AM (2.5 μM) for 30 min at 37 °C. After washing three times with PBS, the cells were observed by CLSM (Ex = 488 nm, Em = 530 nm).

**Intracellular CO Level:** 4T1 cells were seeded into confocal dishes (5.0 × 10^4^ cells per dish) and incubated overnight. Subsequently, the cells were treated with the cells were treated with (I) PBS, (II) MC@HMOS, (III) HMOS@Au, (IV) MC@HMOS@Au, or (V) MC@HMOS@Au@RGD (*C*_nanoparticles_ = 50 μg/mL) for 12 h. Next, the mixture of PdCl_2_ and FLCO-1 (1:1) was added and incubated for 20 min. Subsequently, washed the cells three times with PBS and used an inverted microscope to obtain the fluorescence images.

**Detection of ROS generation in Cells:** 4T1 cells were seeded into confocal dishes (5.0 × 10^4^ cells per dish) and incubated for 24 h. Thereafter, the cells were treated with (I) PBS, (II) MC@HMOS, (III) HMOS@Au, (IV) MC@HMOS@Au, or (V) MC@HMOS@Au@RGD (*C*_nanoparticles_ = 50 μg/mL) for 12 h. The treated cells were then stained with DCFH-DA (10.0 μM) for 30 min at 37 °C in the dark. After washing three times with PBS, the cells were observed by CLSM.

**Monitoring the Changes of Mitochondrial Membrane Potential:** 4T1 cells were seeded into confocal dishes and incubated for 24 h to adhere. After that, the cells were treated with (I) PBS, (II) MC@HMOS, (III) HMOS@Au, (IV) MC@HMOS@Au, or (V) MC@HMOS@Au@RGD (*C*_nanoparticles_ = 50 μg/mL) for 12 h. Afterward, the cells were stained by JC-1 (10 μg/mL), and imaged *via* CLSM (JC-1 monomers, Ex = 514 nm, Em = 529 nm; JC-1 aggregates, Ex = 585 nm, Em = 590 nm).

**Detection of MDA Level in Cells:** 4T1 cells were first seeded into six-well plates (2.0 × 10^5^ cells/well), and allowed to adhere overnight at 37 °C. The culture medium was then replaced with a fresh one (1.0 mL) containing (I) PBS, (II) MC@HMOS, (III) HMOS@Au, (IV) MC@HMOS@Au, or (V) MC@HMOS@Au@RGD (*C*_nanoparticles_ = 50 μg/mL). After incubation for further 12 h, cells were then collected and homogenized in RIPA buffer for 30 min at 4 °C. Cell lysates were then collected *via* centrifugation at 12 000 rpm for 10 min, and the protein concentration was determined using a BCA kit. MDA levels in 4T1 cells were determined according to the manufacturer’s protocols.

**Cytotoxicity Assay:** HUVECs or U87 cells were seeded into 96-well plates (5.0 × 10^3^ cells/well) and incubated overnight at 37 °C. After washing with PBS, the cells were treated with MC@HMOS@Au@RGD with varying concentrations of nanoparticles (*C*_nanoparticles_ = 3.13-100 μg/mL). Then, 10 μL of methyl thiazolyl tetrazolium (MTT, 5.0 mg/mL) was added into each well. After an additional 4.0 h of incubation, the culture media was removed, and then 100 μL of DMSO was added to the cells. The absorbance was recorded at a wavelength of 570 nm using a microplate reader (Biotek Synergy H1).

Meanwhile, the cytotoxicity of different formulations was determined by MTT assay. Briefly, 4T1 cells were seeded into 96-well plates (5.0 × 10^3^ cells/well) and incubated overnight at 37 °C. Then, the culture medium was replaced with the fresh DMEM containing MC@HMOS, HMOS@Au, MC@HMOS@Au, or MC@HMOS@Au@RGD at different concentrations (*C*_nanoparticles_ = 3.13-100 μg/mL). After co-incubation for 24 h, the relative cell viability was measured according to the above similar procedure.

**Tumor Model:** All animal procedures were performed following the Guidelines for Care and Use of Laboratory Animals of Southern Medical University, and approved by the Animal Ethics Committee of Southern Medical University. The assigned approval/accreditation number is SCXK(YUE)2021-0041. To establish the tumor models, female Balb/c mice (5 weeks old, 15-20 g) were subcutaneously implanted with 4.0 × 10^6^ of 4T1 cells in the right back side. The sizes of tumors were measured using a vernier caliper, and the tumor volumes were calculated as follows: tumor volume (mm^3^) = width^2^ × length/2.

**Pharmacokinetic Analysis:** 4T1 tumor-bearing mice were intravenously injected with around 100 µL of MC@HMOS@Au@RGD (dosage = 5.0 mg/kg) after the tumor volume reached ~ 120 mm^3^. 20 µL of venous blood in the orbit of mice was collected at certain time points. The Mn concentration in the obtained blood was measured by ICP-OES after a series of pretreatments.

***In Vitro* and *In Vivo* Ultrasound (US) Imaging Performance:** For *in vitro* US imaging, various solutions (MC@HMOS@Au@RGD, MC@HMOS@Au@RGD + H_2_O_2_, MC@HMOS@Au@RGD + H_2_O_2_+ GSH) were added to a transparent gel tube and then monitored via a Vevo LAZR imaging system (Visual Sonics Inc., Toronto, ON, Canada). For *in vivo* US imaging, MC@HMOS@Au@RGD (10 mg/mL, 100 µL) were intravenously administered into 4T1 tumor-bearing mice (n=3). Prior to the injection as well as at certain intervals (1.0, 3.0, 6.0, and 12 h), B-mode images were captured using the US system of EPIQ 7 (Philips, Netherlands).

**Biodistribution Analysis:** 4T1-bearing mice (n = 3) were intravenously injected with MC@HMOS@Au@RGD (dosage = 5.0 mg/kg). At 1.0, 3.0, or 6.0 h post-injection, the mice were sacrificed to collect their major organs including heart, liver, spleen, lung, kidney, and tumors for biodistribution analysis *via* the ICP-OES measurements.

***In Vivo* Antitumor Effect:** When the tumor volume reached 80-100 mm^3^, 4T1 tumor-bearing mice were randomly divided into 5 groups. The various groups of mice were intravenously injected with (I) PBS, (II) MC@HMOS, (III) HMOS@Au, (IV) MC@HMOS@Au, or (V) MC@HMOS@Au@RGD (dosage of 5.0 mg/kg). The administration was conducted on the day 1 and 7. The tumor volume and body weight were recorded every other day.

In another parallel groups, on the second day of treatments, mice were euthanized, and the major organs (heart, liver, spleen, lung, kidney, and tumors) were collected, fixed in 4.0 % paraformaldehyde, and stained with hematoxylin and eosin (H&E). At the same time, terminal deoxynucleotidyl transferase (TdT)-mediated deoxyuridine triphosphate (dUTP)-biotin nick-end labeling (TUNEL) staining was also performed to further evaluate the therapeutic effect of different formulations.

***In Vivo* Biocompatibility and Biosafety:** Hemolysis experiment was performed to examine the hemocompatibility of the MC@HMOS@Au@RGD. Briefly, 1.5 mL of blood collected from the inner canthus vein plexus of mice was diluted with 3.5 mL of normal saline (NS), followed by centrifugation and washing to obtain the pure red blood cells (RBCs) suspended in 5.0 mL of NS solution. Thereafter, 100 μL of RBC suspension was mixed with 900 μL NS (negative control), water (positive control), and NS solutions of MC@HMOS@Au@RGD with different concentrations (25.0, 50.0, 100, 200, or 400 μg/mL), respectively. After 2.0 h incubation at 37 ^o^C, all the samples were centrifuged at 20000 × g) for 10 min. Then, the photos of the samples were taken, and the absorbance of the obtained supernatants at 540 nm was measured *via* a UV-vis spectrophotometer. The hemolysis rate was calculated according to the following formula: hemolysis rate (%) = [(A(sample, 540 nm)－A(negative, 540 nm))/(A(positive, 540 nm)－A(negative, 540 nm))] × 100%.

Besides, healthy Balb/c mice were injected intravenously with MC@HMOS@Au@RGD at a dosage of 5.0 or 10.0 mg/kg and sacrificed at 3.0 days post-injection. The blood sample of each mouse was collected and stabilized with heparin for blood routine (including red blood cell (RBC), white blood cell (WBC), platelet (PLT), hematocrit (HCT), lymphocytes (LYM), hemoglobin (HGB)) or blood biochemical (including alanine aminotransferase (ALT), aspartate aminotransferase (AST), alkaline phosphatase (ALP) and creatinine (CREA)) analysis.

**Statistical Analysis:** The experimental data are presented as mean ± S.D. The experiments were performed at least in triplicate, and data were analyzed with the SPSS software using one-way ANOVA followed by a post hoc Tukey’s test. The significance level was fixed as * *P* < 0.05, ** *P* < 0.01, *** *P* < 0.001, or **** *P* < 0.0001.


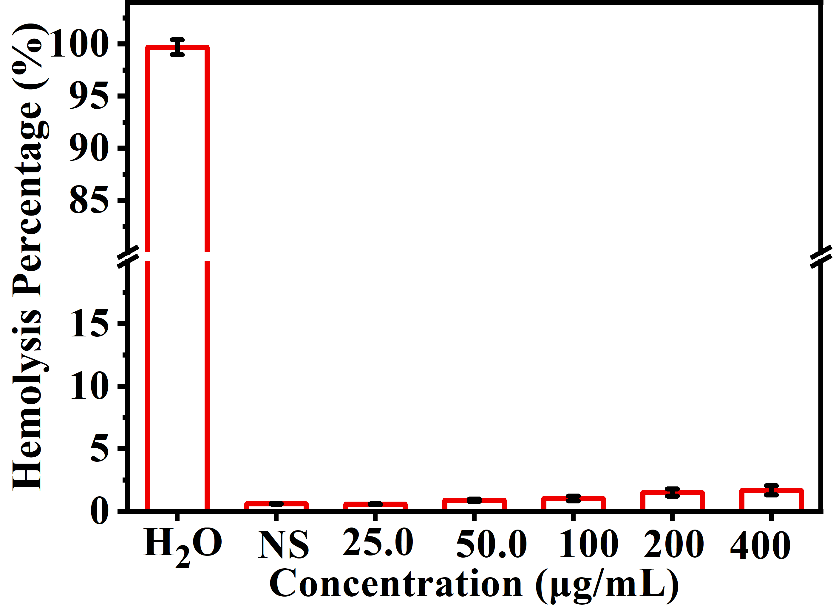


**Figure S12.** Hemolysis percentage of the mouse RBCs treated with pure water, normal saline (NS), or MC@HMOS@Au@RGD with various concentrations at 37.0 °C for 4.0 h (n = 3).


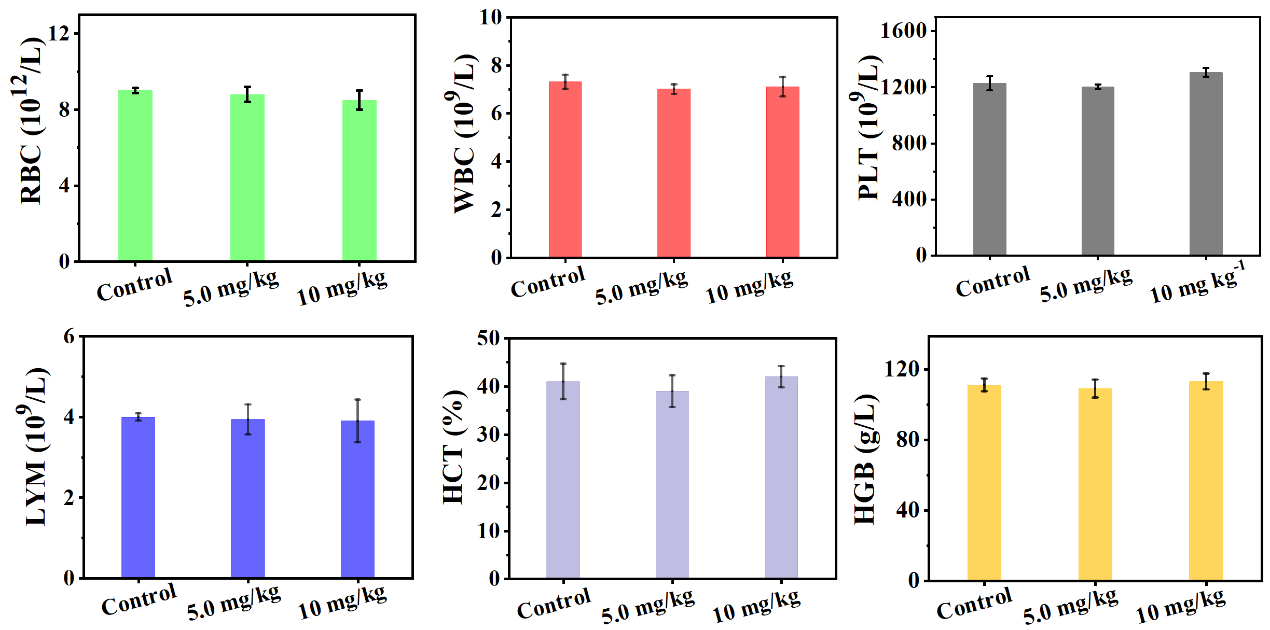


**Figure S2.** Hematological index (such as RBC, WBC, PLT, LYM, HCT, and HGB) measurements of mice on the 3rd day after intravenous injection of MC@HMOS@Au@RGD with a dosage of 5.0 or 10.0 mg/kg. Mean ± S.D., n = 3.


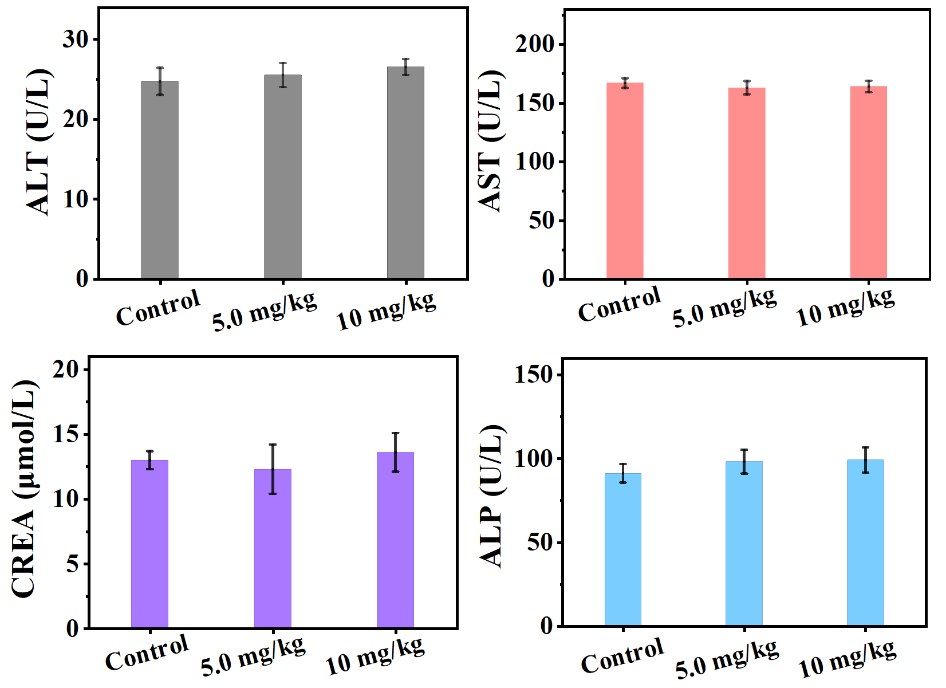


**Figure S3.** Biochemical analysis of mice serum on the 3rd day after intravenous injection of MC@HMOS@Au@RGD with a dosage of 5.0 or 10.0 mg/kg, including ALT, AST, CREA, and ALP. Mean ± S.D., n = 3.


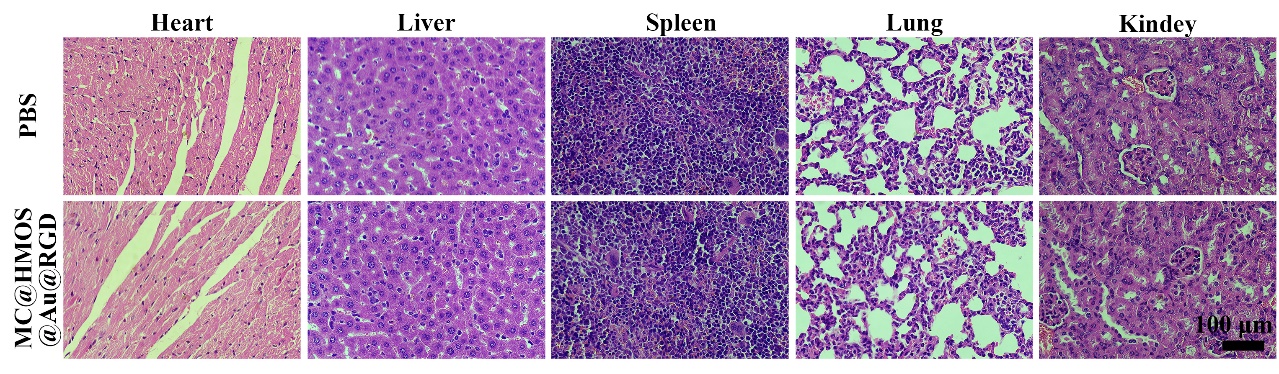


**Figure S4.** Representative optical microscopic pictures of the H&E-stained major organs (heart, liver, spleen, lung, and kidney) from the 4T1 tumor-bearing mice after various treatments with PBS or MC@HMOS@Au@RGD.
